# Neuroprotective Effects of Palmatine via the Enhancement of Antioxidant Defense and Small Heat Shock Protein Expression in A*β*-Transgenic *Caenorhabditis elegans*

**DOI:** 10.1155/2021/9966223

**Published:** 2021-09-15

**Authors:** Weizhang Jia, Qina Su, Qiong Cheng, Qiong Peng, Aimin Qiao, Xiongming Luo, Jing Zhang, Ying Wang

**Affiliations:** ^1^School of Biosciences & Biopharmaceutics, Guangdong Province Key Laboratory for Biotechnology Drug Candidates, Guangdong Pharmaceutical University, Guangzhou 510006, China; ^2^Institutes for Life Sciences and School of Medicine, South China University of Technology, Guangzhou 510641, China

## Abstract

Palmatine is a naturally occurring isoquinoline alkaloid that has been reported to display neuroprotective effects against amyloid-*β*- (A*β*-) induced neurotoxicity. However, the mechanisms underlying the neuroprotective activities of palmatine remain poorly characterized *in vivo*. We employed transgenic *Caenorhabditis elegans* models containing human A*β*_1-42_ to investigate the effects and possible mechanisms of palmatine-mediated neuroprotection. Treatment with palmatine significantly delayed the paralytic process and reduced the elevated reactive oxygen species levels in A*β*-transgenic *C. elegans*. In addition, it increased oxidative stress resistance without affecting the lifespan of wild-type *C. elegans*. Pathway analysis suggested that the differentially expressed genes were related mainly to aging, detoxification, and lipid metabolism. Real-time PCR indicated that resistance-related genes such as *sod-3* and *shsp* were significantly upregulated, while the lipid metabolism-related gene *fat-5* was downregulated. Further studies demonstrated that the inhibitory effects of palmatine on A*β* toxicity were attributable to the free radical-scavenging capacity and that the upregulated expression of resistance-related genes, especially *shsp*, whose expression was regulated by HSF-1, played crucial roles in protecting cells from A*β*-induced toxicity. The research showed that there were significantly fewer A*β* deposits in transgenic CL2006 nematodes treated with palmatine than in control nematodes. In addition, our study found that A*β*-induced toxicity was accompanied by dysregulation of lipid metabolism, leading to excessive fat accumulation in A*β*-transgenic CL4176 nematodes. The alleviation of lipid disorder by palmatine should be attributed not only to the reduction in fat synthesis but also to the inhibition of A*β* aggregation and toxicity, which jointly maintained metabolic homeostasis. This study provides new insights into the *in vivo* neuroprotective effects of palmatine against A*β* aggregation and toxicity and provides valuable targets for the prevention and treatment of AD.

## 1. Introduction

Once there is an imbalance between the production and clearance of amyloid-*β* peptide (A*β*), the accumulation of A*β* initiates self-assembly and the self-assembled A*β* then turns into toxic oligomers, large A*β* fibrils, and plaques associated with the onset and progression of Alzheimer's disease (AD) [1, 2]. Considerable research evidence suggests that oxidative stress is an early event in the development of AD, preceding the classic formation of fibrils that are eventually deposited as insoluble A*β* plaques and neurofibrillary tangles [3, 4]. Meanwhile, the aggregation and deposition of A*β* further increase oxidative stress and aggravate the inflammatory response, thereby causing progressive damage to neurons [5]. Therefore, the complex pathological mechanisms of AD include the aggregation of monomeric A*β* into oligomers or fibrils and A*β*-mediated oxidative stress. Prevention of these processes requires the regulation of signaling pathways to inhibit A*β* aggregation and excessive free radical release in order to maintain cellular homeostasis. Heat shock factor 1 (HSF-1) is an essential regulator of both proteotoxicity and aging [6]. Small heat shock proteins (sHSP), which constitute one type of HSP regulated by HSF-1, act as the front line of defense for preventing or reversing abnormal protein aggregation [7, 8]. More importantly, some of them have been found to exert neuroprotective functions. Specifically, they interact with misfolded and damaging protein aggregates, such as A*β*, in AD to reduce the accumulation of misfolded proteins, block oxidative stress, and attenuate neuroinflammation and neuronal apoptosis; thus, they hold great potential as promising therapeutic agents in neurodegenerative diseases [9, 10]. Oxidative damage is a common and prominent feature of various neurodegenerative diseases and is implicated in the pathogenesis of AD [11]. Studies have shown that AD and other neurodegenerative diseases are associated with elevated levels of oxidative stress biomarkers and impaired antioxidant defense systems in the brain and peripheral tissues [12]. Active compounds with antioxidant properties exert neuroprotective effects by augmenting antioxidant defense and inhibiting A*β*-induced toxicity, which can normalize biomarkers related to oxidant/antioxidant imbalance [13–15].

Although organisms can respond to endogenous and exogenous stressors continuously and attempt to maintain homeostasis, they need externally supplied active substances, particularly when they are facing persistent and overwhelming stress, to adjust the molecular network in order to rebuild a steady state [16]. Otherwise, diseases can occur. Natural products, such as alkaloids, polyphenols, and saponins, have a variety of pharmacological activities [17, 18]. The neuroprotective activity of natural products that can inhibit A*β* aggregation and toxicity by inducing antioxidant and anti-inflammatory responses, regulating stress-related signaling, and modulating A*β* production and clearance has been extensively studied and used in the prevention and treatment of AD [19]. Alkaloids, which form a class of natural nitrogen-containing secondary metabolites, are important active components in Chinese Herbal Medicines. These compounds exert neuroprotective effects through suppression of oxidative stress, neuroinflammation, and apoptosis; reduction of A*β* aggregation; and enhancement of A*β* clearance. Through these effects, the compounds improve functional outcomes in AD [20–23]. Thus, they have great application value for the development of therapeutic agents for the treatment of AD. Although studies have confirmed that many alkaloids potentially exhibit anti-AD effects *in vitro* and *in vivo*, the molecular mechanisms responsible for these effects still need further study.

Palmatine, an isoquinoline alkaloid, has been reported to possess extensive biological functions, such as antioxidant, anti-inflammatory, neuroprotective, and blood lipid-regulating functions [21, 24]. In particular, studies have shown that palmatine might display anti-AD effects by inhibiting the activity of cholinesterase, decreasing A*β* aggregation, reducing the generation of high levels of reactive oxygen species (ROS), and attenuating oxidative damage [24–27]. Nevertheless, little is known about the inhibitory effects of palmatine on A*β* aggregation and toxicity *in vivo* and the signaling pathways that exert the neuroprotective effects of palmatine are also not well understood. Due to advantageous features such as a short lifespan, rapid generation time, tractable genetic manipulation, and fully sequenced genome [28], the model species *Caenorhabditis elegans* has been widely used to study aging and aging-related neurodegenerative diseases and was therefore employed to investigate the action mechanisms of palmatine-mediated neuroprotective effects. The experiments indicated that palmatine inhibits A*β* aggregation and toxicity by enhancing antioxidant defense and sHSP expression to maintain homeostasis in *C. elegans*. This study provides insights into the neuroprotective effects of palmatine in vivo and provides valuable targets for the prevention and treatment of AD.

## 2. Materials and Methods

### 2.1. Chemical and Materials

The isopropyl-beta-d-thiogalactopyranoside (IPTG), 2′,7′-dichlorofluorescein diacetate (DCFH-DA), 5-fluoro-2′-deoxyuridine (FUDR), cholesterol, and 1,1′-dimethyl-4,4′-bipyridinium dichloride (Paraquat or PQ) were purchased from Sigma Chemical Corp. (St. Louis, MO, USA). The detection assay kits of SOD and CAT enzyme activity and protein quantification (Bicinchoninic acid (BCA)) were acquired from Beyotime (Shanghai, China). The palmatine, Oil red O, Sudan black B, and Thioflavin S (ThS) were obtained from Aladdin (Shanghai, China). We bought the RNA extraction reagent (TRIzol) from Invitrogen (Carlsbad, CA, USA). The DNase I, restriction enzymes XbaI and KpnI, plasmid preparation, reverse transcription, and real-time PCR kits were provided by TaKaRa (Dalian, China). Other chemical reagents used in this study were supplied by Tianjin Damao Chemical Reagent Factory (Tianjin, China).

### 2.2. *C. elegans* and Culture

There are several worm strains involved in our work: the wild-type N2, the A*β*-transgenic CL2006 *{dvIs2 [pCL12(unc-54/human Abeta(1-42) minigene) + rol-6(su1006)]}* and CL4176 *{dvIs27 [myo-3p::Abeta(1-42)::let-851 3*′*UTR) + rol-6(su1006)]}*, the transgenic CF1553 containing *sod-3p*::GFP *{muIs84 [(pAD76) sod-3p::GFP + rol-6(su1006)]},* and CL2070 containing *hsp-16.2p*::GFP *{dvIs70 [hsp-16.2p::GFP + rol-6(su1006)]}*. This work was approved by the experimental animal ethics committee of Guangdong Pharmaceutical University with the approval number gdpulac2019015. *Escherichia coli*, such as OP50, NA22, and HT115 strains, were selected to feed worms based on different experimental conditions. All *C. elegans* were provided by the Caenorhabditis Genetics Center. The A*β*-transgenic CL2006 and CL4176 worms and the wild-type N2, CF1553, and CL2070 worms were cultured and maintained on nematode-growing medium (NGM) plates at 15°C and 20°C, respectively. The synchronous population was prepared by treatment of gravid adults with alkaline hypochlorite and hatched overnight.

### 2.3. Food Clearance and Body Length Assays

To select the suitable concentration range of palmatine, the wild-type nematodes were used to conduct food clearance and body length assays. In food clearance, 20 *μ*L of S medium containing approximately 20 L1-stage worms was put into 80 *μ*L of S medium including NA22 and palmatine with indicated concentration (0.05, 0.1, 0.2, and 0.4 mM) in a 96-well microplate and cultured at 20°C. Absorbance value (570 nm) was measured every 24 h and continued for six days. For the body length, L1-stage worms were placed on a NGM plate fed with OP50 containing different concentrations of palmatine (0.05, 0.1, 0.2, and 0.4 mM) and cultured at 20°C for two days. The worm images (approximately 100 per group) were acquired and analyzed using a Mshot MF52 inverted microscope (Mingmei, Guangzhou, China) with digital software.

### 2.4. Paralytic Assays

Using A*β*-transgenic CL2006 and CL4176 strains, we preliminarily detected the effect of palmatine for inhibiting A*β*-toxicity according to previously described [29]. The L1-stage CL2006 worms were fed on NGM solid plates including OP50 and different concentrations of palmatine (0.1 and 0.2 mM), which were placed at 15°C for 45 h and shifted to the new NGM solid plates (approximately 100 per group) containing OP50 with FUDR (75 *μ*g/mL) and different concentrations of palmatine (0.1 and 0.2 mM) at 20°C for inducing A*β* peptide expression. Paralyzed worms were counted every day until all were palsied. For the CL4176 strain, the L1-stage worms were cultured at 15°C for 36 h on NGM plates containing different concentrations of palmatine (0.1 and 0.2 mM) and shifted to 23°C for inducing A*β* peptide expression. The amount of paralyzed worms was counted every 2 h until all palsied.

### 2.5. Measurement of the ROS Level

The ROS level in worms was evaluated through the DCFH-DA method as described previously [30]. L1-stage CL4176 worms were cultured at 15°C for 36 h on a NGM solid plate with or without palmatine (0.2 mM) and shifted to 23°C for another 36 h. Approximately 2000 worms in each group were lysed in PBST buffer (PBS containing 0.1% Tween 20) and used to collect the supernatant by centrifugation at 10000 g for 5 min. The protein content was determined by a BCA protein assay kit. A total volume of 50 *μ*L DCFH-DA (100 mM) was placed into a black 96-well microplate containing 50 *μ*L supernatant. The intensity of DCF was calculated with the Synergy H1 Microplate Reader (BioTek, Dallas, TX, USA) at 488 nm of excitation and 525 nm of emission.

### 2.6. Oxidative Survival and Lifespan Assays

Oxidative stress caused by paraquat was performed with the wild-type worms as reported previously [31]. L1-stage worms were incubated in S medium fed with NA22 at 20°C until the L4 stage and put into a 96-well microplate containing NA22, ampicillin (100 *μ*g/mL), and FUDR (75 *μ*g/mL). The worms were further cultured with or without palmatine (0.2 mM) for one day at 20°C and then exposed to paraquat (75 mM). The survival was counted every 12 h until all were dead (approximately 100 worms per group). For the lifespan assay, L4-stage wild-type worms were put into a 96-well microplate at the density of 15–20 individuals per well in 100 *μ*L of culture medium (approximately 100 worms per group) and treated with or without palmatine (0.2 mM). The part of worms alive was counted every two days until all were dead.

### 2.7. Transcriptome Analysis and Real-Time PCR Verification

L1-stage wild-type worms were cultured on NGM plates fed OP50 with or without palmatine (0.2 mM) for two days at 20°C, and then, the worms were collected. Total RNA was prepared from the worms by using TRIzol, and further purified mRNA was used for Illumina sequencing at Shanghai Majorbio Bio-pharm Technology Co. Ltd. (China). A threshold false discovery rate (FDR) (≤ 0.05) and fold change (≥2.0) were used as criteria to screen differentially expressed genes (DEGs), which were categorized on the basis of Kyoto Encyclopedia of Genes and Genomes (KEGG) pathway analysis. At the same time, samples were prepared for real-time PCR detection as described above and the primer sequences are listed in Table 1.

### 2.8. Measurement of sod-3 and hsp-16.2 Expressions

Transgenic CF1553 and CL2070 worms containing *sod-3p*::GFP and *hsp-16.2p*::GFP reporters were used to determine the expression of the *sod-3* and *hsp-16.2* genes. Transgenic worms were cultured with or without palmatine (0.2 mM) on NGM plates at 20°C for two days and anesthetized on microscope slides for observation and determination of *sod-3p*::GFP and *hsp-16.2p*::GFP expression. Images were captured by using an Mshot MF52 inverted fluorescence microscope coupled with digital software, and the expression of the respective genes was ascertained by assessing the GFP signal using ImageJ software.

### 2.9. Detection of *SOD* and *CAT* Activity Levels

The wild-type strain was synchronized as described above, and L1-stage worms were cultured at 20°C for two days on NGM solid plates with or without palmatine (0.2 mM). Approximately 2000 worms were gathered and lysed in PBST buffer. The activity levels of SOD and CAT were detected with SOD and CAT assay kits, respectively, and a BCA protein assay kit was used to determine the protein concentration.

### 2.10. RNA Interference

In an RNAi-mediated gene knockdown experiment, *E. coli* HT115 was chosen as a food source for the worms as previously described with some changes [30]. An RNAi plasmid was built by partially cloning the *hsf-1* cDNA sequence into the *L4440* empty vector. The primer sequences with restriction sites for XbaI (TCTAGA) and KpnI (GGTACC) are displayed in Table 1. L1-stage CL2006 worms were cultured with or without palmatine at 15°C on NGM solid plates seeded with HT115 containing the *L4440* empty plasmid or the *hsf-1* recombinant plasmid for 45 h. The worms (approximately 100 per group) were then transferred to NGM plates and fed HT115 (containing the *L4440* empty plasmid or the *hsf-1* recombinant plasmid) in the presence of 5-FUdR (75 *μ*g/mL) and palmatine (0.2 mM) at 20°C to induce A*β* peptide expression. Worm paralysis was observed, and the paralyzed worms were counted microscopically every day until all worms were palsied.

### 2.11. ThS Staining Assay

The wild-type and A*β*-transgenic CL2006 strains were used for the assay as described previously [32]. Here, wild-type worms were stained as negative control. L1-stage A*β*-transgenic CL2006 worms were cultured at 20°C for 90 h on NGM plates with or without palmatine (0.2 mM). In another group, CL2006 worms were continuously cultured at 15°C for 90 h and used for comparative analysis. All worms were collected and fixed at 4°C for one day in 4% paraformaldehyde (PFA). After PFA fixation, the worms were incubated at 37°C for one day in permeabilization solution including *β*-mercaptoethanol (5%), Triton X-100 (1%), and Tris−HCl (125 mM). The worms were washed and soaked in ThS solution (containing 0.125% ThS and 50% ethanol) at ambient temperature for 2 min and then decolorized with 50% ethanol until the staining agent in the solution completely disappeared. Images of the anterior pharyngeal bulb were acquired by using an Mshot MF52 inverted microscope.

### 2.12. Fat Staining

Fat staining was performed by using Oil Red O and Sudan black B as previously described [33, 34]. Briefly, L1-stage N2 and CL4176 strains were cultured on solid NGM plates seeded with OP50 with or without palmatine (0.2 mM). The N2 worms were cultured at 20°C for three days, while the CL4176 worms were cultured at 15°C for 36 h and shifted to 23°C for another 36 h. The worms used for Oil Red O staining were fixed at 4°C for 30 min in 4% PFA and subjected to three freeze-thaw cycles. The worms were dehydrated for 15 min in 60% isopropanol and stained for 6 h in an Oil Red O staining solution containing 40% water and 60% Oil Red O stock solution. For Sudan black B staining, the worms were first fixed in 4% PFA, subjected to 3 freeze-thaw cycles, dehydrated in 25, 50, and 70% ethanol, and then dyed for 12 h in a 50% saturated Sudan Black B solution (70% ethanol). The images were obtained by using an Mshot MF52 inverted microscope, and ImageJ software was used for quantitative analysis.

### 2.13. Statistical Analysis

The data were analyzed by using GraphPad Prism version 7.0 for Windows (San Diego, California, USA). The *t*-test and one-way analysis of variance (ANOVA) were selected as the analytical methods. The survival and lifespan curves were created with the Kaplan-Meier method, and a log-rank test was selected to evaluate the statistical significance. The primers were designed by using Primer 3 version 0.4.0 software, and *β-actin* was used as the reference gene. All experiments were carried out at least three times, and a *p* value of <0.05 was considered to indicate statistical significance.

## 3. Results

### 3.1. Inhibition of A*β* Toxicity by Palmatine in A*β*-transgenic Nematodes

**Figure 1:**
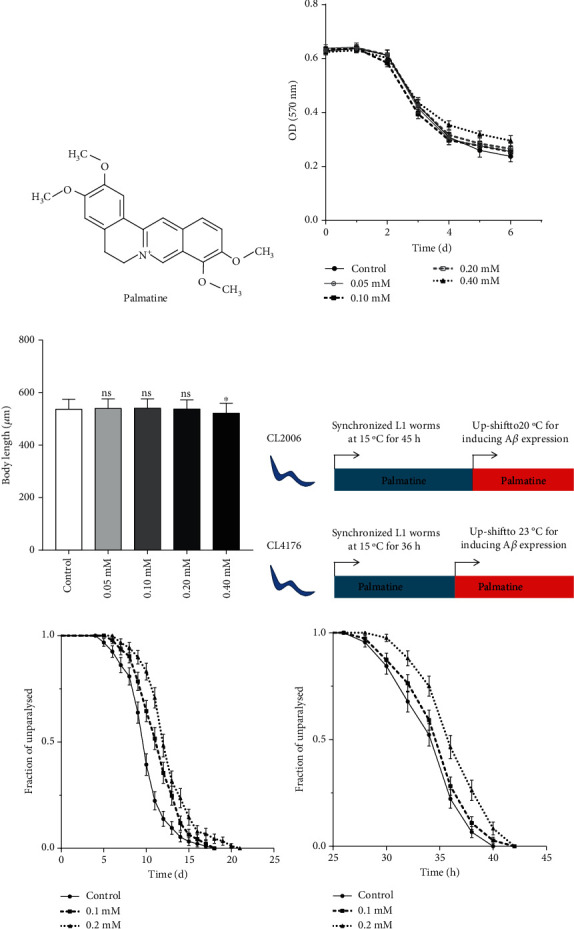
Inhibition of A*β*-toxicity of palmatine in transgenic *C. elegans*. (a) Chemical structure of palmatine. (b) Food clearance. Synchronized wild-type worms were cultured at 20°C in a 96-well plate with or without palmatine at different concentrations. The absorbance (at 570 nm) was detected daily for six days using a microplate reader. The results were displayed as mean ± SD from 5 parallel wells. (c) Body length. Synchronized wild-type worms were cultured at 20°C in NGM plates with or without palmatine at different concentrations for two days. The results were displayed as mean ± SD of approximately 100 worms. Statistical analysis was carried out with a one-way ANOVA. ns: no significant difference; ^∗^*p* < 0.05. (d) The experimental flowchart in A*β*-transgenic CL2006 and CL4176 nematodes. (e) The paralytic assay of CL2006 nematodes. Synchronized L1-stage worms were cultured with or without palmatine for 45 h at 15°C and upshifted to 20°C for inducing A*β* peptide expression. The fraction of paralytic worms was counted every day until all were palsied. (f) The paralytic assay of CL4176 nematodes. Synchronized L1-stage worms were cultured for 36 h at 15°C in NGM plates with or without palmatine and upshifted to 23°C for inducing A*β* peptide expression. The fraction of paralytic worms was counted every 2 h until all were palsied. The results were displayed as Kaplan-Meier survival curves and analyzed by using a log-rank test.

*C*. *elegans* is a powerful model organism with which to research the molecular mechanisms of neurodegenerative diseases and screen effective neuroprotective drugs [28]. In this work, we studied the neuroprotective effects of palmatine in the A*β*-transgenic *C. elegans* CL2006 and CL4176 strains (Figure 1(a)), which express human A*β*_1-42_ in constitutive and inducible manners and show progressive and rapid paralytic phenotypes, respectively. The concentration of palmatine was confirmed through food clearance and body length assays, which indicated that a concentration of ≤0.2 mM was suitable in wild-type worms (Figures 1(b) and 1(c)). Therefore, according to the experimental design in Figure 1(d), we preliminarily tested the effects of palmatine against A*β* toxicity at concentrations of 0.1 and 0.2 mM. As shown in Figure 1(e), palmatine at 0.1 and 0.2 mM effectively protected against A*β* toxicity in progressively paralyzed CL2006 nematodes. A beneficial effect of palmatine was also observed in rapidly paralyzed CL4176 nematodes (Figure 1(f)). It is worth noting that palmatine at 0.2 mM showed a more effective protective effect against A*β* toxicity than palmatine at 0.1 mM and significantly delayed the paralytic process. In summary, these results suggest that palmatine exhibits neuroprotective activity against A*β* toxicity in A*β*-transgenic nematode models.

### 3.2. Alleviation of Oxidative Stress Rather Than Prolongation of Lifespan

**Figure 2:**
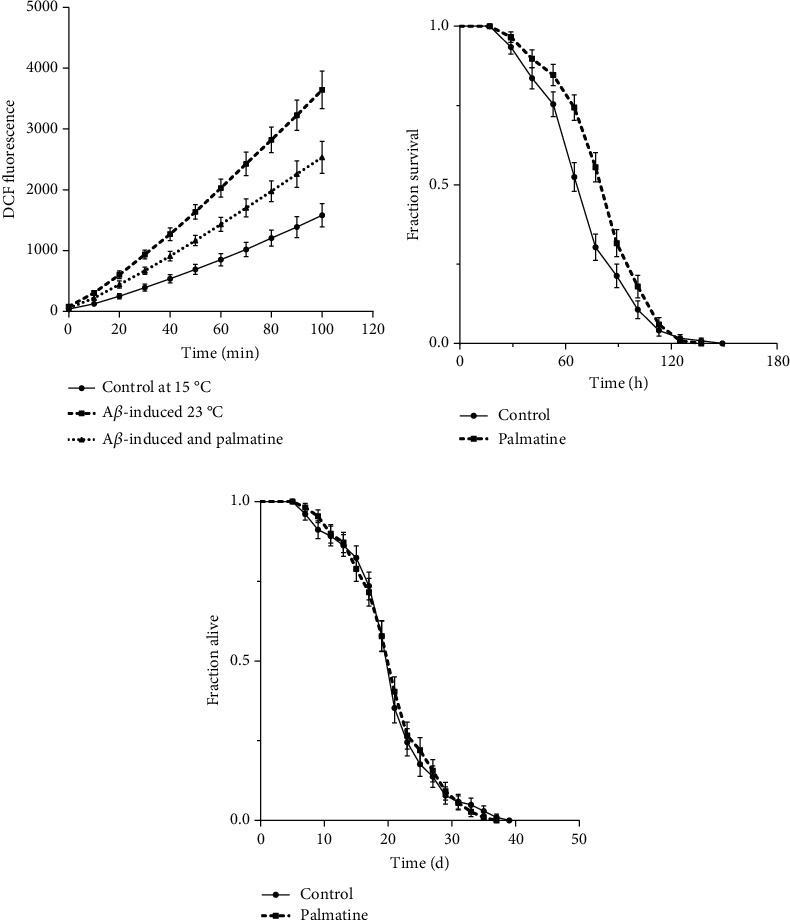
The effects of palmatine on the ROS level, oxidative stress, and lifespan. (a) The effects of palmatine on ROS in the AD model CL4176 strain. Synchronized *L1-stage* CL4176 worms were cultured with or without palmatine (0.2 mM) for 36 h at 15°C and then cultured at 23°C for another 36 h. These worms were collected and homogenized, and then, the lysate was used for detection of the ROS level. The curve is from a single experiment (values are means ± SD, *n* = 3). (b) Oxidative survival assay in wild-type nematodes. L4-stage wild-type nematodes (approximately 100 for each group) were pretreated with or without palmatine at 20°C for 24 h and then treated with 75 mM paraquat, and the fraction of survival was scored every 12 h until all dead. (c) Lifespan assay in wild-type nematodes. L4-stage wild-type worms were put into a 96-well plate at the density of 15–20 individuals/well in 100 *μ*L of culture medium (≈100 worms/group) and administered with or without palmatine (0.2 mM). The fraction of alive worms was counted every two days until all deaths. The results were displayed as Kaplan-Meier survival curves and analyzed by using a log-rank test.

There is considerable evidence to suggest that A*β* toxicity increases oxidative stress and free radical formation, which are closely associated with the progression of AD [11]. Since palmatine inhibited A*β* toxicity *in vivo*, as demonstrated above, we investigated the effects of palmatine on the oxidative status using transgenic CL4176 nematodes. As shown in Figure 2(a), the ROS level was significantly higher in the homogenate of A*β*-induced worms at 23°C than in the homogenate of the control worms at 15°C. However, the ROS level was significantly lower in the homogenate of worms treated with palmatine at 23°C than in the homogenate of the untreated worms. These data suggest that palmatine plays a positive role in ROS scavenging and improves stress tolerance in A*β*-transgenic CL4176 worms. Next, we detected the protective effects of palmatine against oxidative stress through the assessment of paraquat-induced oxidative damage in wild-type nematodes. In Figure 2(b), the survival of the worms treated with palmatine prior to paraquat damage was significantly higher than that of the control worms, indicating the antioxidative stress activity of palmatine. However, as shown in Figure 2(c), palmatine did not significantly change the lifespan of the worms. Taken together, these findings show that the ROS-reducing capability of palmatine may contribute to its protection against A*β* toxicity.

### 3.3. Transcriptome-Based Discovery of Genes and Pathways Related to the Effects of Palmatine against A*β* Toxicity

**Figure 3:**
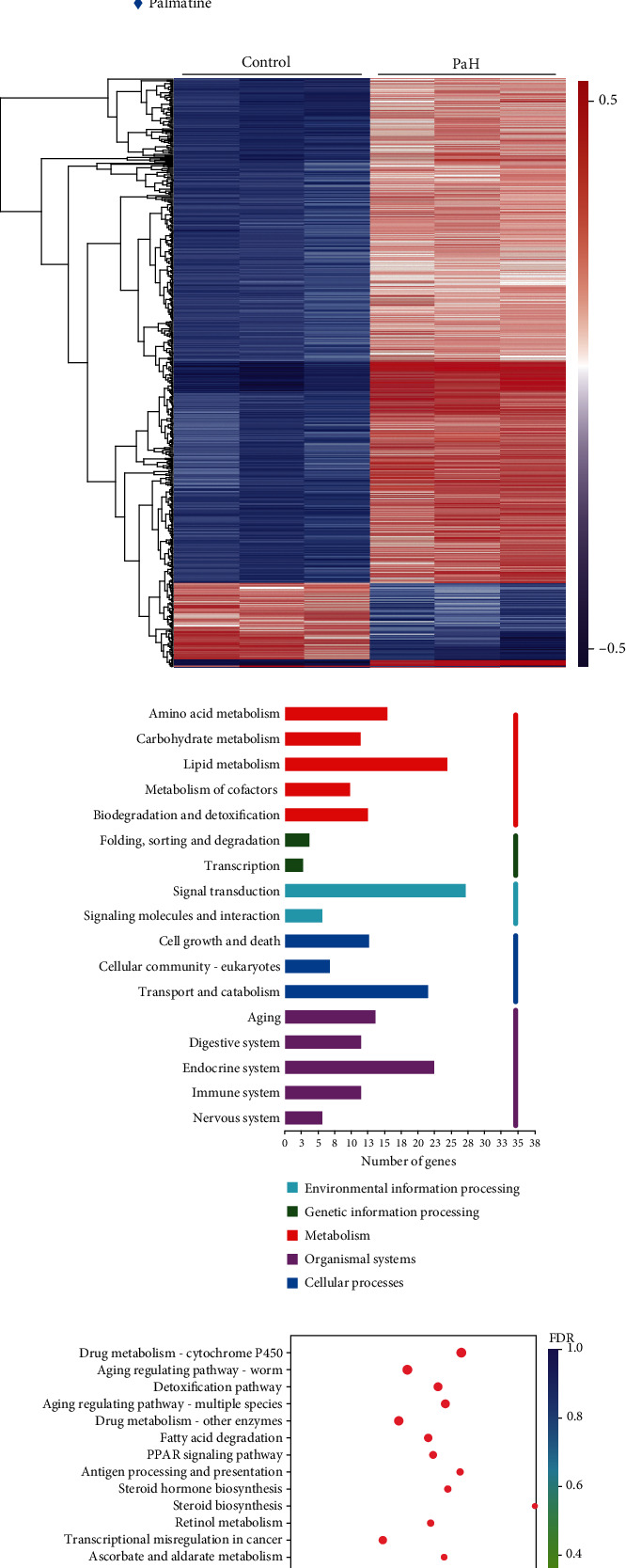
Transcriptome analysis of *C. elegans* treated with palmatine. (a) Principal component analysis (PCA) analysis. (b) The heatmap analysis of DEGs. (c) The annotation of DEGs in the palmatine-treated group classified on the basis of KEGG pathway analysis. (d) The significantly enriched DEG pathway. Synchronized L1-stage wild-type worms were cultured at 20 °C for two days with or without palmatine (0.2 mM) on NGM plates seeded with *OP50*, and three independent biological replicates for each group were collected for transcriptome sequencing.

Gene expression profiles can be used as signatures to search for the signaling pathways and downstream target genes of active compounds [35]. To understand the transcriptomic responses to palmatine, transcriptome sequencing was carried out on the Illumina MiSeq platform for wild-type nematodes treated with or without palmatine (0.2 mM) for 48 h. The raw RNA-Seq reads reported in this paper are found in the NCBI Sequence Read Archive (accession number PRJNA667515). Principal component analysis (PCA) was used to assess the clustering of the samples, which manifested an obvious distinction between the control and palmatine-treated groups (Figure 3(a)). After treatment with palmatine, 602 DEGs were detected, including 522 upregulated genes and 80 downregulated genes. In Figure 3(b), the patterns of the changes in transcript abundance are shown in the heatmap of the DEGs, which were identified using an FDR cutoff of 0.05 and a fold change threshold of 2. To retrieve the functional information on the DEGs in the experimental group, coregulated genes were further classified into different categories, including lipid metabolism, biodegradation and detoxification, signal transduction, transport and catabolism, and aging of the endocrine system (Figure 3(c)). Furthermore, in pathway enrichment analysis (Figure 3(d)), the genes regulated by palmatine treatment were associated mainly with the aging regulation pathway, detoxification, and fatty acid degradation.

### 3.4. Validation of Gene Expression Levels by Real-time PCR

**Figure 4:**
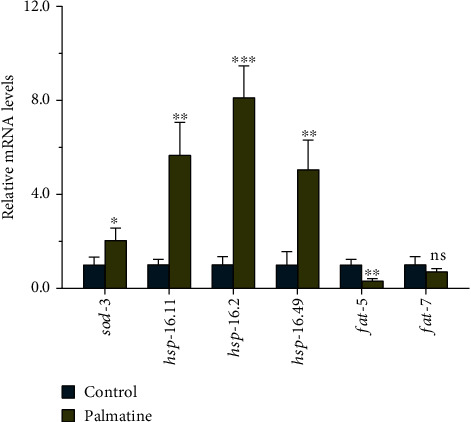
The verification of indicated target genes from RNA-Seq analysis by qPCR. L1-stage wild-type worms were cultured at 20°C for 48 h on NGM plates with or without palmatine (0.2 mM). Three independent biological replicates for each group were collected for real-time PCR. The result is presented as mean ± SD, and the statistical analysis was carried out with unpaired *t*-test. ns: no significant difference; ^∗^*p* < 0.05; ^∗∗^*p* < 0.01; ^∗∗∗^*p* < 0.001.

Among the RNA-sequencing data, we focused on the data for some signaling pathways related to stress resistance, such as aging, detoxification, and lipid metabolism pathways. To further validate the gene expression profiles, the gene expression levels of six representative candidate genes were analyzed via real-time PCR. The regulation patterns for the selected genes, including *sod-3*, *hsp-16.11*, *hsp-16.2*, *hsp-16.49*, and *fat-5*, were consistent with the RNA-sequencing data; only marginal differences in the relative fold changes were observed. However, the *fat-7* gene did not show statistically significant differences among the control and palmatine-treated groups. As shown in Figure 4, special attention should be given to the *shsp* genes, which were significantly upregulated in the worms treated with palmatine compared with the normal control worms. Overall, the findings show that palmatine treatment can activate resistance-related genes to inhibit A*β* toxicity.

### 3.5. Enhancement of sod-3p::GFP Expression and SOD and CAT Activity Levels

**Figure 5:**
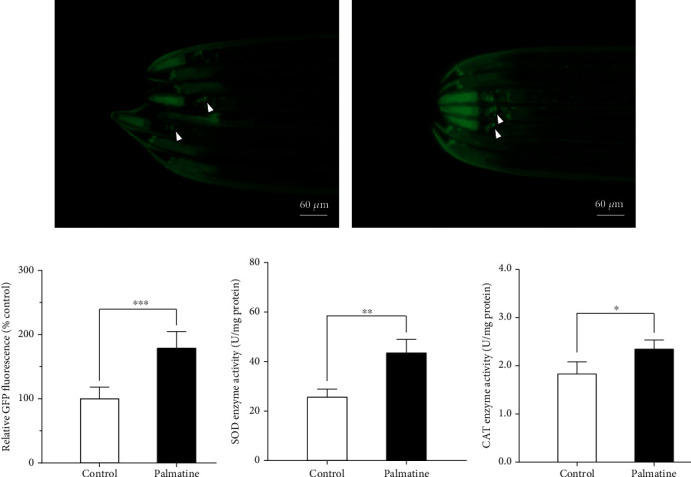
The effects of palmatine on *sod-3p*::GFP expression, SOD, and CAT activities. (a, b) The images of *sod-3p*::GFP treated with or without palmatine (0.2 mM). (c) Quantification of *sod-3p*::GFP fluorescence intensity. The transgenic CF1553 strain was cultured at 20°C for two days on NGM plates with or without palmatine (0.2 mM). The images were obtained by using a Mshot MF52 inverted fluorescence microscope. Quantified *sod-3p*::GFP intensity was done by ImageJ software. The result was displayed as mean ± SD (*n* = 20–25), and the statistical analysis was carried out with unpaired *t*-test. ^∗∗∗^*p* < 0.001. (d) The effect of palmatine on the enzyme activity of SOD in wild-type worms. (e) The effect of palmatine on the enzyme activity of CAT in wild-type worms. *W*ild-type *worm*s were cultured from L1- to L4-stage at 20°C on NGM plates with or without palmatine (0.2 mM), and then, the worms were lysed to detect the enzyme activities of SOD and CAT. The result was displayed as mean ± SD, and the statistical analysis was done by unpaired *t*-test. ^∗^*p* < 0.05; ^∗∗^*p* < 0.01.

Since SOD is a key antioxidant enzyme for ROS scavenging, we employed the CF1553 strain containing a *sod-3p*::GFP transgenic reporter to explore the effects of palmatine on the transcriptional change of the *sod-3* gene. When treated with palmatine for two days, the fluorescence level of *sod-3p*::GFP in the worms fed palmatine was markedly higher than that in the normal control worms, indicating that palmatine has the ability to enhance the expression of *sod-3* in this nematode strain (Figures 5(a)–5(c)). In addition, we measured the enzyme activity of SOD in wild-type worms fed with or without palmatine (0.2 mM). As shown in Figure 5(d), the level of the SOD enzyme was significantly higher in the worms fed palmatine than in the normal control worms, further demonstrating that palmatine upregulated SOD expression. In addition, CAT is an essential antioxidant enzyme that can prevent cells from being poisoned by H_2_O_2_; we further examined the enzyme activity of CAT in wild-type worms fed palmatine (0.2 mM). As presented in Figure 5(e), the level of the CAT enzyme was significantly higher in the worms fed palmatine than in the normal control worms. In summary, these results indicate that palmatine has the ability to enhance antioxidant defense against oxidative stress caused by endogenous or exogenous stressors.

### 3.6. Involvement of the HSF-1 Transcription Factor in the Inhibition of A*β* Toxicity by Palmatine

**Figure 6:**
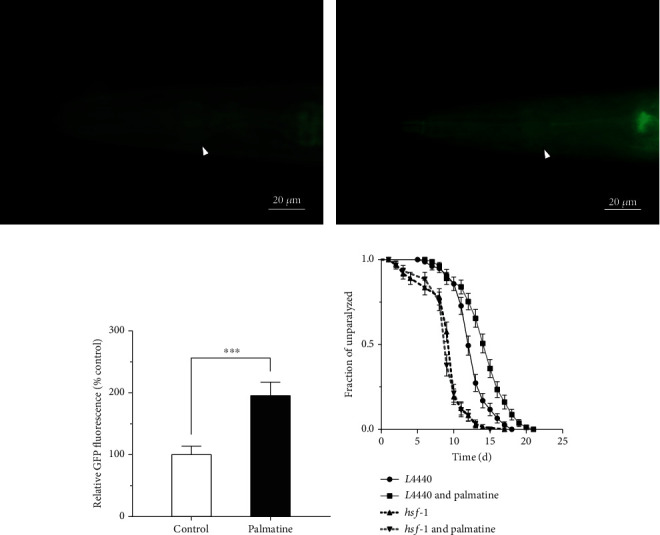
The effects of palmatine on *hsp-16.2p*::GFP expression and HSF-1 signaling pathway. (a, b) The images of *hsp-16.2p*::GFP in control and palmatine-treated groups. (c) Quantification of *hsp-16.2p*::GFP fluorescence intensity. The transgenic CL2070 strain was cultured at 20°C for two days on NGM plates with or without palmatine (0.2 mM). The images were obtained by using a Mshot MF52 inverted fluorescence microscope. Quantified *hsp-16.2p*::GFP intensity was done by ImageJ software. The result was displayed as mean ± SD (*n* = 20–25), and the statistical analysis was carried out with unpaired *t*-test. ^∗∗∗^*p* < 0.001. (d) RNAi analysis of the change in inhibition of A*β*-toxicity by palmatine. L1-stage CL2006 strain was treated with or without palmatine (0.2 mM) at 15°C on NGM plates containing HT115 with *L4440* empty plasmid or *hsf-1* recombinant plasmid for 45 h and upshifted to 20°C for inducing A*β* peptide expression. The fraction of paralytic worm was counted every day until all were palsied. The result was displayed as the Kaplan-Meier survival curve and analyzed by using a log-rank test.

There is abundant evidence that the enhancement of protein folding and antiapoptotic capacity induced by elevations in HSP levels has the potential to improve therapeutic efficacy in neurodegenerative diseases [36, 37]. As shown by the transcriptome-based discovery of genes and pathways, the induced expression of sHSP may play the most important role in the inhibition of A*β* toxicity by palmatine. Therefore, we used the transgenic CL2070 strain expressing an *hsp-16.2p*::GFP transgenic reporter to explore the effects of palmatine on the transcriptional change of the *hsp-16.2* gene. After treatment with palmatine for two days, the fluorescence level of *hsp-16.2p*::GFP in the worms fed palmatine was significantly higher than that in the normal control worms, indicating that palmatine enhanced the expression of *hsp-16.2* in this worm model (Figures 6(a)–6(c)). It is well known that the transcription factor HSF-1 coordinately activates the expression of *shsp* genes [36]. Thus, we further determined the protective effects of palmatine in *C. elegans* after *hsf-1* RNAi to assess whether its inhibition of A*β* toxicity involved HSF-1. As presented in Figure 6(d), palmatine significantly alleviated the toxicity induced by A*β* when HT115 bacteria transformed with the empty vector *L4440* were used, yet the alleviation of A*β*-induced toxicity disappeared when recombinant *hsf-1* RNAi bacteria were used, indicating that the inhibition of A*β* toxicity by palmatine was mainly dependent on HSF-1. In summary, these results suggest that palmatine-mediated suppression of A*β*-induced toxicity relies on the regulator HSF-1 and subsequent upregulation of the expression of its target genes, such as *hsp-12.11*, *hsp-16.2*, and *hsp-16.49*.

### 3.7. Reductions in A*β* Deposits

**Figure 7:**
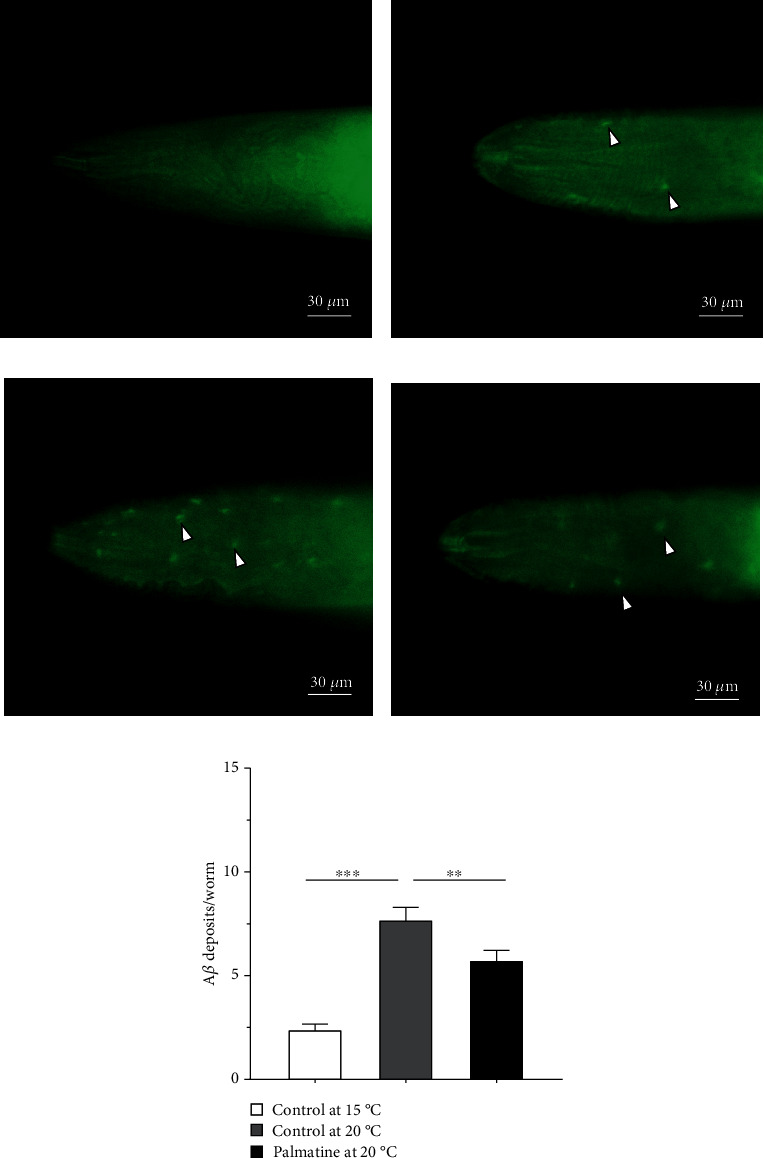
The effects of palmatine on A*β* deposits. (a) The image of wild-type nematodes. The worms were cultured at 20°C for 90 h and stained by ThS as negative control. (b) The images of A*β*-transgenic CL2006 nematodes at 15°C. The nematodes were continuously cultured at 15°C for 90 h and used for comparative analysis. (c, d) The images of A*β*-transgenic CL2006 nematodes treated with or without palmatine. The nematodes were incubated with or without palmatine (0.2 mM) at 20°C for 90 h. (d) Quantification of A*β* deposits. All the worms were collected and stained by ThS staining, and the fluorescence images were obtained by using a Mshot MF52 inverted fluorescence microscope. The depositions of A*β* were quantified after counting the ThS-positive deposits in the anterior pharyngeal bulb in each worm. The result was displayed as mean ± SD, and the statistical analysis was done by using a one-way ANOVA. ^∗∗^*p* < 0.05; ^∗∗∗^*p* < 0.001.

To investigate the inhibitory effects of palmatine on A*β* aggregation, ThS staining was performed in transgenic CL2006 nematodes to detect A*β* deposition, and then, the A*β* deposits in the worm head region were counted. Consistent with previous findings [32], the wild-type nematodes, used as negative controls, lacked ThS fluorescence because of the lack of A*β* deposition (Figure 7(a)). When A*β*-transgenic CL2006 worms were continuously cultured at 15°C, small amounts of A*β* deposits were formed due to the constitutive expression of the A*β* peptide in this strain (Figure 7(b)). After the worms were shifted to 20°C to induce A*β* peptide expression, the number of A*β* deposits per nematode was significantly increased compared to that in the worms incubated at 15°C, suggesting that elevated temperature can significantly enhance the expression of the A*β* peptide (Figure 7(c)). Compared to that in untreated CL2006 nematodes, A*β* deposition in nematodes treated with palmatine was obviously inhibited in the A*β*-transgenic worms (Figure 7(d)). The quantification of A*β* deposits also revealed significantly fewer deposits in palmatine-treated worms than in untreated worms (Figure 7(e)). Combined with the above experimental results indicating that palmatine can effectively delay the paralytic process of the CL2006 strain, these results indicate that palmatine can effectively inhibit A*β* aggregation and alleviate A*β*-induced toxicity.

### 3.8. Mitigation of the A*β*-mediated Lipid Metabolism Disorder

**Figure 8:**
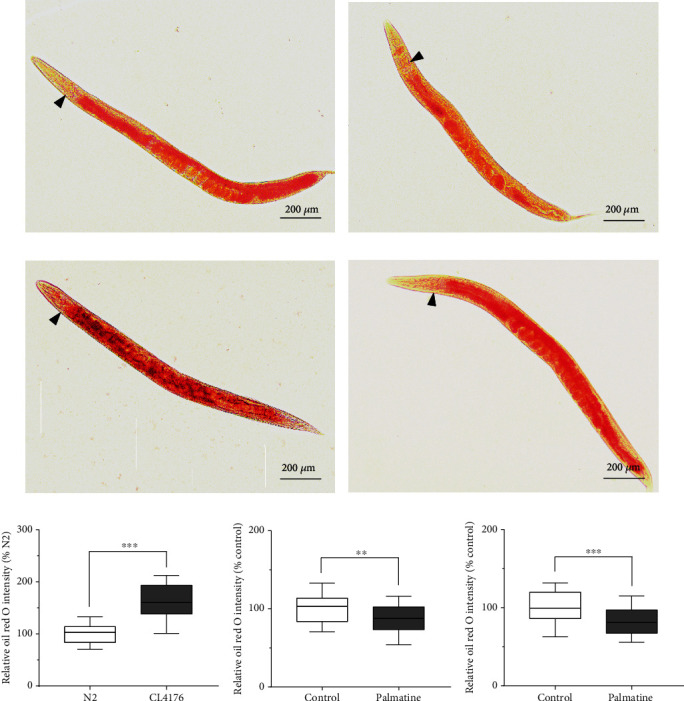
The effects of palmatine on fat accumulation. (a, b) The optical images upon Oil Red O staining in wild-type worms treated with or without palmatine (0.2 mM). (c, d) The optical images upon Oil Red O staining in CL4176 worms treated with or without palmatine (0.2 mM). (e) Comparative analysis of the Oil Red O intensity in wild-type and CL4176 worms treated with or without palmatine (0.2 mM). (f) Quantitative analysis of the Oil Red O intensity in wild-type worms treated with or without palmatine (0.2 mM). (g) Quantitative analysis of the Oil Red O intensity in CL4176 worms treated with or without palmatine (0.2 mM). L1-stage wild-type strain was cultured at 20°C for three days on NGM plates with or without palmatine (0.2 mM). Meanwhile, L1-stage CL4176 strain was cultured at 15°C for 36 h on NGM plates containing with or without palmatine (0.2 mM) and then placed at 23°C for another 36 h. The wild-type and CL4176 worms were collected and used for Oil Red O staining, respectively. The images were obtained by using a Mshot MF52 inverted fluorescence microscope. Quantified intensities were performed using ImageJ software. The results were displayed as mean ± SD (*n* = 30), and the statistical analyses were done by using an unpaired *t*-test. ^∗^*p* < 0.05; ^∗∗∗^*p* < 0.001.

Since transcriptome analysis suggested that lipid synthesis was obviously repressed by palmatine treatment, we used Oil Red O and Sudan Black B staining to detect the fat content in intestinal and hypodermal cells in wild-type and transgenic CL4176 nematodes (Figures 8(a)–8(d), staining with Oil Red O; Figure S1(a), S1(b), S1(c) and S1(d), staining with Sudan Black B) [33, 34]. Surprisingly, the content of lipid droplets was significantly increased after A*β* peptide expression was induced in transgenic CL4176 worms compared with normal wild-type worms (Figure 8(e), staining with Oil Red O; Figure S1(e), staining with Sudan Black B), suggesting that A*β* aggregation and toxicity were accompanied by dysregulated lipid metabolism that caused excessive fat accumulation in A*β*-transgenic CL4176 nematodes. In addition, the effects of palmatine on fat accumulation in wild-type and transgenic CL4176 strains were evaluated. The palmatine-treated nematodes exhibited a lower lipid content than the wild-type nematodes (Figure 8(f), staining with Oil Red O; Figure S1(f), staining with Sudan Black B). Supplementation with palmatine in A*β*-transgenic CL4176 nematodes also significantly decreased the lipid content, as revealed by Oil Red O and Sudan Black B staining (Figure 8(g) and Figure S1(g)). These results indicate that palmatine reduces fat synthesis in wild-type nematodes and effectively inhibits lipid metabolism disorder induced by A*β* aggregation and toxicity in transgenic CL4176 nematodes.

## 4. Discussion

Although there is continuing debate about the A*β* hypothesis, the accumulated evidence supports the idea that steady-state imbalance between the production and clearance of A*β* is one of the important initiating events in the development of AD [38]. Elevated levels of A*β* oligomers have been found to drive the development of oxidative stress, neuroinflammation, synaptic loss, and nerve cell death [4]. Therefore, there is considerable interest in finding active compounds that can reduce A*β* aggregation and alleviate A*β*-induced toxicity [19, 21, 39]. Alkaloids are a large group of structurally complex natural products that exhibit a wide range of biological effects, including antioxidant and anti-inflammatory effects and A*β* production- and clearance-regulated effects [40–42]. In the present study, we confirmed that palmatine, a naturally occurring isoquinoline alkaloid, significantly suppressed A*β*-induced paralysis and displayed neuroprotective effects in the A*β*-transgenic CL2006 and CL4176 strains. The inhibition of the paralytic process was related to a reduction in A*β* oligomerization and alleviation of A*β*-induced toxicity [32]. Therefore, if there are similar mechanisms in humans and other species, the protective effects of palmatine against A*β* aggregation and toxicity may provide a rationale for its beneficial effects in humans.

The hallmarks of AD, such as A*β* oligomerization and neurofibrillary tangle formation, are intertwined with excessive ROS levels and elevated oxidative stress, which are considered to be the common effectors of the cascade of degenerative events [14, 43]. Previous research has shown that palmatine has the potential to inhibit oxidative stress by scavenging free radicals [27, 42]. In our study, palmatine reduced the elevated ROS levels derived from A*β* toxicity in the CL4176 strain. It is known that abiotic stresses, such as exposure to paraquat, often cause oxidative stress damage in organisms via overproduction of ROS [30, 31]. In the paraquat-induced oxidative damage model, we further demonstrated that palmatine treatment enhances resistance to oxidative stress. These results suggest that palmatine has an antioxidant effect against A*β* toxicity by reducing ROS production. However, palmatine did not affect the lifespan of wild-type nematodes, indicating that the protective mechanisms against oxidative stress may be distinct from mechanisms that delay senescence.

By transcriptome sequencing, we obtained candidate pathways and genes related to the effects of palmatine, including genes/pathways related to aging, detoxification, and lipid metabolism. In particular, the results highlighted that the expression of *shsp* was significantly upregulated by palmatine. These genes play important roles in modulating A*β* aggregation and toxicity [9, 10]. In addition, antioxidant-related genes, such as *sod-3*, were also activated, which may have enhanced antioxidant defense to protect cells from oxidative damage [44]. Palmatine has been shown to reduce the ROS level in the transgenic CL4176 strain and alleviate the oxidative damage caused by paraquat-induced oxidative stress. The elimination of excessive ROS is mediated by a range of antioxidant defense systems, which require the participation of antioxidant enzymes such as SOD and CAT [44, 45]. Our study confirmed that palmatine increases the fluorescence intensity of *sod-3p*::GFP in transgenic CF1553 nematodes and the activities of SOD and CAT in wild-type nematodes. These results agree with previous findings that alkaloids might activate the cellular antioxidant defense system against oxidative insults [20, 46, 47]. Together, these studies suggest that palmatine reduces the oxidative stress caused by A*β* toxicity by eliminating ROS and indicate a correlation between the enhancement of antioxidant defense and the alleviation of A*β* toxicity in transgenic nematodes.

Protein aggregation is one of the important characteristics of neurodegenerative diseases such as AD [43, 48]. HSP family members are the key cellular components that maintain protein homeostasis under unfavorable conditions, which might effectively block the formation of protein aggregates and prevent the occurrence and development of neurodegenerative diseases [10, 49]. Studies have indicated that a lack of sHSP aggravates the AD phenotype, whereas increased expression improves the symptoms of AD [50]. In the present study, palmatine upregulated the expression of s*hsp* genes, including *hsp-16.11*, *hsp-16.2*, and *hsp-16.49*, in wild-type nematodes and enhanced the *hsp-16.2p*::GFP fluorescence intensity in transgenic CL2070 nematodes. As such, it is possible that the increased expression of sHSP suppressed protein aggregation and alleviated A*β* toxicity, suggesting that sHSP plays important roles in the neuroprotective effects of palmatine. It is known that the expression of sHSP is primarily determined by the transcription factor HSF-1, while decreased activity of HSF-1 has been shown to be a key part of the deleterious cascade in neurodegenerative disease [36, 37]. Our study showed that knockdown of *hsf-1* using RNAi not only significantly accelerated the process of paralysis but also completely abolished palmatine-induced inhibition of A*β* toxicity in transgenic CL2006 nematodes. These results are in accordance with previous studies showing that HSF-1 participates in the inhibition of A*β* toxicity [6, 37] and suggest that the increased resistance of worms treated with palmatine is closely related to HSF-1. Therefore, palmatine-mediated inhibition of A*β* toxicity involves the regulator HSF-1 and regulation of the expression of its target genes, such as *hsp-16.11*, *hsp-16.2*, and *hsp-16.49*.

Research has indicated that alkaloids have the potential to inhibit and prevent AD through both cholinesterase and A*β* pathways and through improvement of antioxidant capacity [20–23, 42]. It is worth noting that unlike the activation of antioxidant defense to alleviate A*β*-induced oxidative stress, the palmatine-induced upregulation of sHSP is associated with the inhibition of A*β* aggregation and breakdown of toxic oligomers [37, 51]. Research has shown the occurrence of a series of morphological transitions of aggregation, including the assembly of A*β* monomers into oligomers and fibrils and the eventual formation of extracellular A*β* plaque deposits [52]. Among the aggregates, A*β* oligomers have been identified as potent cytotoxins that initiate pathological events in AD [5]. Active substances, such as *Ginkgo biloba* extract EGb 761 and ginkgolide A, may convert toxic A*β* oligomers into nontoxic A*β* monomers to delay the paralysis of A*β*-transgenic nematodes [32]. It has been reported that palmatine chloride treatment *in vitro* can suppress the aggregation of tau and break down preformed tau aggregates [53]. In this work, palmatine delayed the process of paralysis and reduced A*β* deposition in transgenic CL2006 nematodes, suggesting that its neuroprotective effects *in vivo* may be achieved via effects on the early stages of the linear pathway, especially the formation of toxic A*β* oligomers.

Palmatine is reported to have the ability to regulate lipid metabolism and to exert anti-obesity effects [24, 27]. In this work, our study found that the aggregation of A*β* in transgenic CL4176 nematodes not only led to a paralytic phenotype but also accompanied excessive fat accumulation caused by A*β* toxicity-induced lipid metabolism disorder. Notably, palmatine markedly decreased the fat content both in the wild-type worms and in the A*β*-transgenic worms with high fat accumulation. A previous study has indicated that oleic acid is the substrate for the production of triacylglycerol and cholesteryl ester and that inhibiting the conversion of saturated stearic acid to oleic acid can contribute to reducing the fat content [54]. The current research showed that palmatine downregulated the expression of a stearoyl-CoA desaturase, *fat-5*, in wild-type worms, which is believed to play a critical role in the transformation of stearic acid into oleic acid [55]. However, overall, considering the modulation of antioxidant defense and sHSP expression, the alleviation of lipid disorder by palmatine is likely attributable not only to the reduction in fat synthesis but also to the inhibition of A*β* aggregation and toxicity, which jointly maintain metabolic homeostasis in transgenic CL4176 nematodes.

## 5. Conclusion

In the present study, palmatine suppressed A*β*-induced paralysis and reduced the elevated ROS levels in A*β*-transgenic nematodes, thus exhibiting neuroprotective effects against A*β* toxicity. Based on DEG analysis, real-time PCR, and verification in transgenic CF1553 and CL2070 strains, our study demonstrated that the inhibition of A*β* toxicity by palmatine was attributable to the free radical-scavenging capacity and to the upregulation of the expression of resistance-related genes such as *sod-3* and *shsp*. In particular, the expression of *shsp*, which requires the involvement of the transcription factor HSF-1, played important roles in protecting cells from A*β* toxicity. In addition, we found that palmatine reduced A*β* deposition and mitigated excessive fat accumulation in the A*β*-transgenic CL2006 and CL4176 strains, respectively. In summary, these studies suggest that palmatine exerts neuroprotective effects through the modulation of antioxidant defense and sHSP expression to support homeostasis in A*β*-transgenic nematodes. The findings provide valuable targets for the prevention and treatment of AD.

## Figures and Tables

**Table 1:** The primer sequences for real-time PCR and RNAi analyses.

Gene	Forward primer (5′ to 3′)	Reverse primer (5′ to 3′)	Application
*β-Actin*	CCACGAGACTTCTTACAACTCCATC	CTTCATGGTTGATGGGGCAAGAG	Real-time PCR
*sod-3*	GAGCTGATGGACACTATTAAGCG	GCACAGGTGGCGATCTTCAAG	Real-time PCR
*hsp-16.11*	CTCCATCTGAATCTTCTGAGATTG	CTTCGGGTAGAAGAATAACACGAG	Real-time PCR
*hsp-16.2*	CTCCATCTGAGTCTTCTGAGATTGT	CTCCTTGGATTGATAGCGTACGA	Real-time PCR
*hsp-16.49*	TCCGACAATATTGGAGAGATTG	GATCGTTTCGAGTATCCATGCT	Real-time PCR
*fat-5*	GTGCTGATGTTCCAGAGGAAGAAC	ATGTAGCGTGGAGGGTGAAGCA	Real-time PCR
*Fat-7*	CCAGAGAAAGCACTATTTCCCAC	CACCAAGTGGCGTGAAGTGT	Real-time PCR
*hsf-1* ^a^	TGCTCTAGACTGTCCCAAGGTGGTCTAACTC	CGGGGTACCTCCCGAATAGTCTTGTTGC	RNAi

^a^Underlines indicate the restriction sites of XbaI (TCTAGA) and KpnI (GGTACC).

## Data Availability

The data that used to support the findings of this work are obtained from the corresponding author on reasonable request.

## Supplementary Materials

Supplementary Materials Figure S1: the effects of palmatine on fat accumulation through Sudan black B staining in the wild-type and CL4176 *C. elegans*.
