# Supplementary material for: Neuroprotective Effects of Palmatine via the Enhancement of Antioxidant Defense and Small Heat Shock Protein Expression in Aβ-Transgenic Caenorhabditis elegans
Source: Oxid Med Cell Longev. 2021 Sep 15;2021:9966223. doi: 10.1155/2021/9966223 (PMC8460366; doi:10.1155/2021/9966223)
Supplement: Supplementary Materials — Figure S1: the effects of palmatine on fat accumulation through Sudan black B staining in the wild-type and CL4176 C. elegans. [file 9966223.f1.doc]

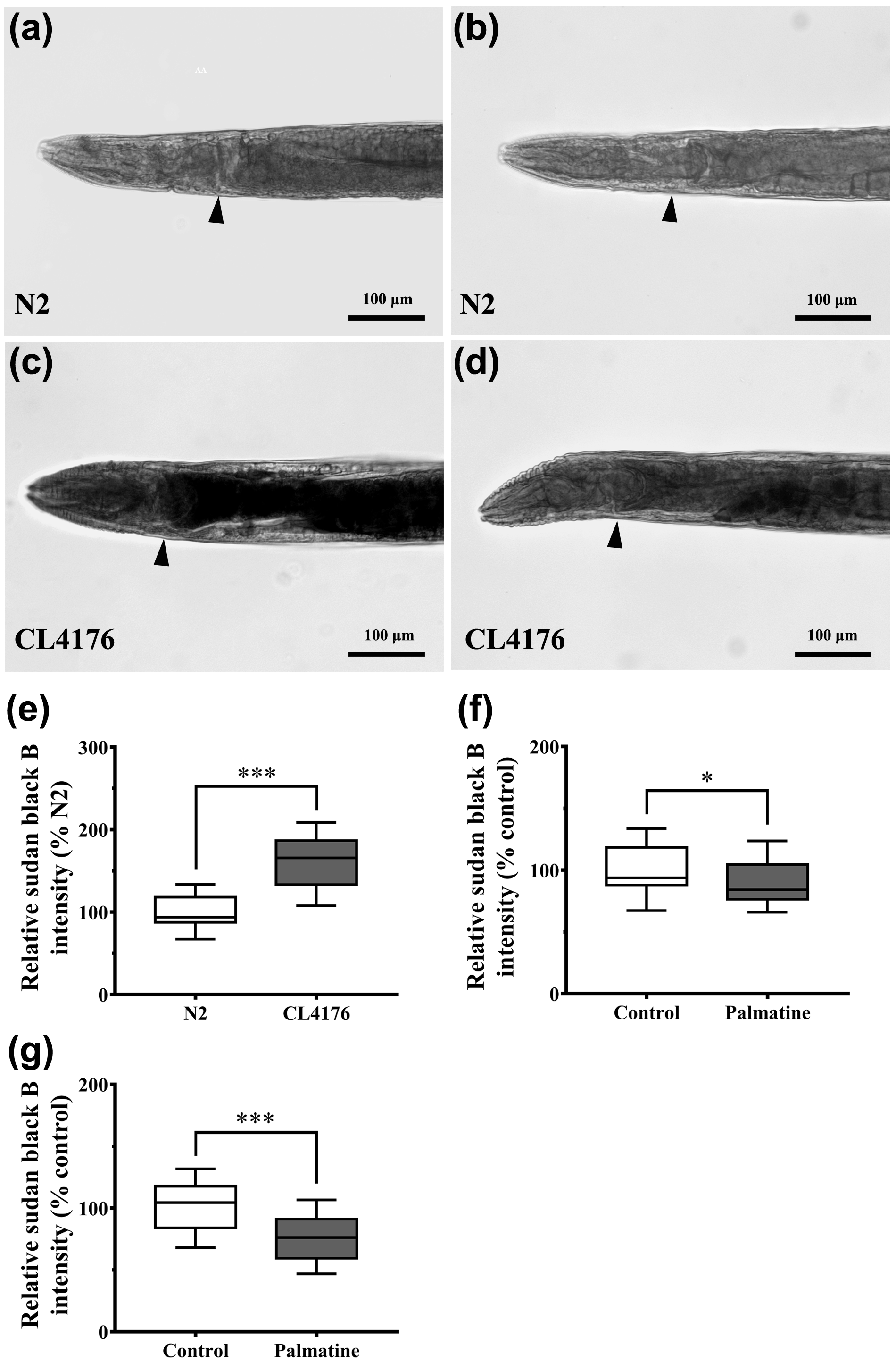


F_IGURE_ S1: The effects of palmatine on fat accumulation through Sudan black B staining in the wild-type and CL4176 *C. elegans*. (a) and (b) The optical images upon Sudan black B staining in the wild-type worms treated with or without palmatine (0.2 mM). (c) and (d) The optical images upon of Sudan black B staining in the CL4176 worms treated with or without palmatine (0.2 mM). (e) Comparative analysis of the Sudan black B intensity in wild-type and CL4176 worms treated with or without palmatine (0.2 mM). (f) Quantitative analysis of the Sudan black B intensity in the wild-type worms treated with or without palmatine (0.2 mM). (g) Quantitative analysis of the Sudan black B intensity in the CL4176 worms treated with or without palmatine (0.2 mM). L1-stage wild-type strain was cultured at 20 °C for three days on NGM plates containing with or without palmatine (0.2 mM). Meanwhile, L1-stage CL4176 strain was cultured at 15 °C for 36 h on NGM plates containing with or without palmatine (0.2 mM), and then placed at 23 °C for another 36 h. The wild-type and CL4176 worms were collected and used for Oil Red O staining, respectively. The images were obained by using a Mshot MF52 inverted fluorescence microscope. Quantified intensities were performed using a Image-J software. The results were displayed as mean ± SD (*n* = 30), and the statistical analyses were done by using a unpaired *t*-test. *: *p* < 0.05; ***: *p* < 0.001.
